# Advanced Value Chain Collaboration in Ghana’s Cocoa Sector: An Entry Point for Integrated Landscape Approaches?

**DOI:** 10.1007/s00267-017-0863-y

**Published:** 2017-04-15

**Authors:** Howard Deans, Mirjam A. F. Ros-Tonen, Mercy Derkyi

**Affiliations:** 1 Independent researcher, London, UK; 2 Department of Geography, Planning and International Development Studies and Centre for Sustainable Development Studies, University of Amsterdam, P.O. Box 15629, Amsterdam, 1001 NC The Netherlands; 3 Department of Forest Science, University of Energy and Natural Resources, Sunyani, Ghana

**Keywords:** Value chain collaboration, public-private-producer partnerships, landscape approaches, cocoa, Ghana

## Abstract

Value chain analyses have focused mainly on collaboration between chain actors, often neglecting collaboration “beyond the chain” with non-chain actors to tackle food security, poverty and sustainability issues in the landscapes in which these value chains are embedded. Comparing conventional and advanced value chain collaborations involving small-scale cocoa farmers in Ghana, this paper analyzes the merits of a more integrated approach toward value chain collaboration. It particularly asks whether advanced value chain collaboration targeting cocoa-producing areas potentially offers an entry point for implementing a landscape approach. The findings detail current chain actors and institutions and show how advanced value chain collaboration has a greater positive impact than conventional value chain collaboration on farmers’ social, human and natural capital. The paper concludes that the integrated approach, focus on learning, and stable relationships with small-scale farmers inherent in advanced value chain collaboration makes it both more sustainable and effective at the local level than conventional approaches. However, its scope and the actors’ jurisdictional powers and self-organization are too limited to be the sole tool in negotiating land use and trade-offs at the landscape level. To evolve as such would require certification beyond the farm level, partnering with other landscape stakeholders, and brokering by bridging organizations.

## Introduction

New forms of value-chain collaboration (VCC) in Ghana’s cocoa sector increasingly target objectives “beyond the chain” such as sustainable sourcing, livelihood improvement, biodiversity enhancement and climate change mitigation (Bitzer 2011; Ros-Tonen et al. 2015). These new forms of VCC are voluntary associations between different actors in the chain that increasingly involve non-chain actors such as non-governmental and (in the case of public–private partnerships) governmental organizations that aim to integrally address multiple objectives in the landscapes in which these chains are embedded (Helmsing and Vellema 2011; Ros-Tonen et al. 2015). In this paper, we refer to such extended partnerships as advanced VCC. This contrasts with conventional VCC or supply chain management, which primarily focuses on vertical value-chain relations and efficiently processing a product through the production cycle without improvements in the process itself (Horvath 2001). Through advanced VCC, companies such as Nestlé, Olam International, and Lindt & Sprüngli AG also invest in direct relationships with farmers by providing inputs (e.g., seeds and fertilizers), training, and/or credit (Ros–Tonen et al. 2015). Hence the alternative term public–private–producer–partnerships (PPPPs or 4Ps) (Thorpe and Maestre 2015). Often, advanced VCCs or PPPPs are driven by corporate social responsibility or, in more recent speak, creating shared values (CSV) motives, which are aimed at simultaneously enhancing a company’s competitiveness and farmers’ economic and social conditions (Porter and Kramer 2011; Kissinger et al. 2013). Companies engage in such partnerships based on the assumption that failing to address societal problems such as food insecurity or unsustainable production will eventually present internal costs to the company in the form of supply failure or productivity losses (Porter and Kramer 2011).

Advanced VCC is an integrated multi-stakeholder approach which affects the landscape level when focusing on sustainable sourcing. This explorative study aims to assess whether this offers a potential opening for implementing a landscape approach. The latter is defined as an integrated, multi-sector, multi-actor and multi-level governance approach toward negotiated trade-offs between different land uses at landscape level to address global challenges such as food insecurity, climate change and biodiversity loss (Sayer et al. 2013, Van Oosten et al. 2014, Ros–Tonen et al. 2014, Reed et al. 2015). Integrated landscape approaches are often externally driven and come with high transaction costs (Hart et al. 2014; Reed et al. 2015, 2016). Hence the importance of finding economically sustainable and locally embedded entry points for the implementation of such approaches. Few studies exist to date that provide insight into such entry points. This paper aims to contribute to this knowledge gap by asking what is the potential of advanced VCC to provide an entry point for implementing landscape approaches.

The next section clarifies some concepts after which we provide context to existing VCCs in the Ghanaian cocoa sector. Next, we elaborate on the methodology employed for this study. The results section then looks at the differences between conventional and advanced VCC as regards actor constellations and institutional arrangements, and their effects on farmers’ social, human, and natural capital. The discussion then addresses the main question by looking at how advanced VCC matches with the principles and enabling factors for landscape approaches. The concluding section synthesizes the findings and makes a suggestion for further research.

## Territorially Embedded Value Chain Collaboration and Landscape Approaches

Value chain studies usually focus on vertical relationships between actors in the chain, such as producers, buyers, traders, retailers, and consumers. These relationships refer to the flow of goods and services from producer to consumer and from design to marketing, concerned with the value added by actors and the resulting income share (Gereffi 1999; Kaplinsky 2000; Ponte 2008).

Further developments in value-chain analysis led to a shift of perspective from the governance of the overall chain, to coordination within specific levels–in this paper the link between a cocoa farmer and local buyer. Subsequently, the focus was not only on vertical but also on horizontal relationships with “flows” including the transfer of knowledge, finance and information (Bolwig et al. 2010). Horizontal analysis goes beyond chain actors directly involved in production or commercialization, enabling a more holistic view to be gained within a specific value chain link (Muradian et al. 2011). This implies that vertical commodity chain relations increasingly merge with horizontal, place-based interactions, contexts, actors and effects (Bolwig et al. 2010; Marsden 2013; Ros-Tonen et al. 2015).

The increasing importance of horizontal relationships results in a growing range of VCCs, with varying degrees of collaboration and coordination between actors (Maertens et al. 2012). Advanced VCC in this paper is characterized by greater integration of smallholders, with the aim of addressing barriers to the chain’s development beyond what is expected within conventional VCCs (Van Wijk and Kwakkenbos 2012). Whilst remaining focused on the commercial gains of collaboration, these advanced VCCs aim for “authentic” or “active” partnerships, characterized by a commitment to relationships, mutual benefits and transparency (De Boer and Tarimo 2012). The broadening playing field of VCC, its multi-stakeholder setting, its embarking on multiple goals and multifunctional landscapes potentially implies increasing opportunities for synergy with landscape approaches. With economic incentives at the heart of VCCs, there is furthermore scope to improve the commercial sustainability of landscape approaches and enhance—often missing—private sector involvement (Milder et al. 2014; Estrada–Carmona et al. 2014).

## The Ghanaian Cocoa Value Chain: Emerging Partnerships in a State-Dominated Sector

Ghana’s cocoa beans are primarily exported for processing into cocoa butter, liquor, and powder for chocolate confectionery, and used in cosmetics and beauty products (Barrientos et al. 2008). Representing a global value chain, cocoa has become the country’s most important agricultural export commodity and a vital contributor to Ghana’s development (Kolavalli and Vigneri 2011; World Bank 2013). The livelihoods of 30% of the population depend upon the cocoa sector (Gockowski et al. 2011). In contrast to other cocoa-producing countries, Ghana only partially liberalized its cocoa market (World Bank 2013). The parastatal Cocoa Board (Cocobod), continues to be a major actor with control throughout the Ghanaian part of the cocoa value chain, setting prices and minimum standards, and licensing buying companies (Laven 2010). It has five subsidiary departments to promote the production, processing, and marketing of cocoa (Cocobod 2015).

With worldwide chocolate sales being projected to increase by 6.2% (Terazono 2014), both the state and cocoa traders have strong interests in investing in the productive capacity of small-scale farmers who are responsible for the majority of Ghana’s cocoa production (Barrientos et al. 2008). The ability to meet a rising demand for both standard and certified cocoa is a challenge, but even the ability to maintain current levels of production faces risks from current low levels of land productivity, an aging farmer population, and a lack of youth entering farming (Barrientos et al. 2008).

Ghana has explicitly promoted partnerships with the private sector (MOFA 2007) to address the need for a sustainable and profitable cocoa economy (World Bank 2013). Examples are partnerships between international cocoa-trading and -processing companies with one or two licensed buying companies, such as Cargill-Akuafo Adamfo; Touton-Produce Buying Company; Kokoopa-Noble Resources; and Lindt-Armajaro Ghana Limited (AGL), which also involves the farmers associated with the buying companies. Such partnerships usually focus on training in good agricultural practices and certification standards; strengthening farmer groups; providing support services and credit to farmers to enable them to rehabilitate their farm, to intensify or diversify; or engaging in schemes for payments for environmental services, reducing emissions from deforestation and forest degradation while enhancing carbon stocks (REDD+), or promoting climate-smart landscapes (Jaskiewicz and Laven 2015). Other VCCs were initiated by the joint action of a government agency and donors (such as the National Cocoa Platform initiated by Cocobod with support of the United Nations Development Program) or by private organizations such as the Cocoa Livelihood Program of the World Cocoa Foundation.

Being a well-established industry that has considerable experience with VCC, the Ghanaian cocoa sector provides an interesting case to analyze the impacts of different kinds of VCCs on farmers’ livelihoods and sustainable practices, and from there assess their potential to align with landscape approaches.

## Methods and Materials

### The Study Area

**Fig. 1:**
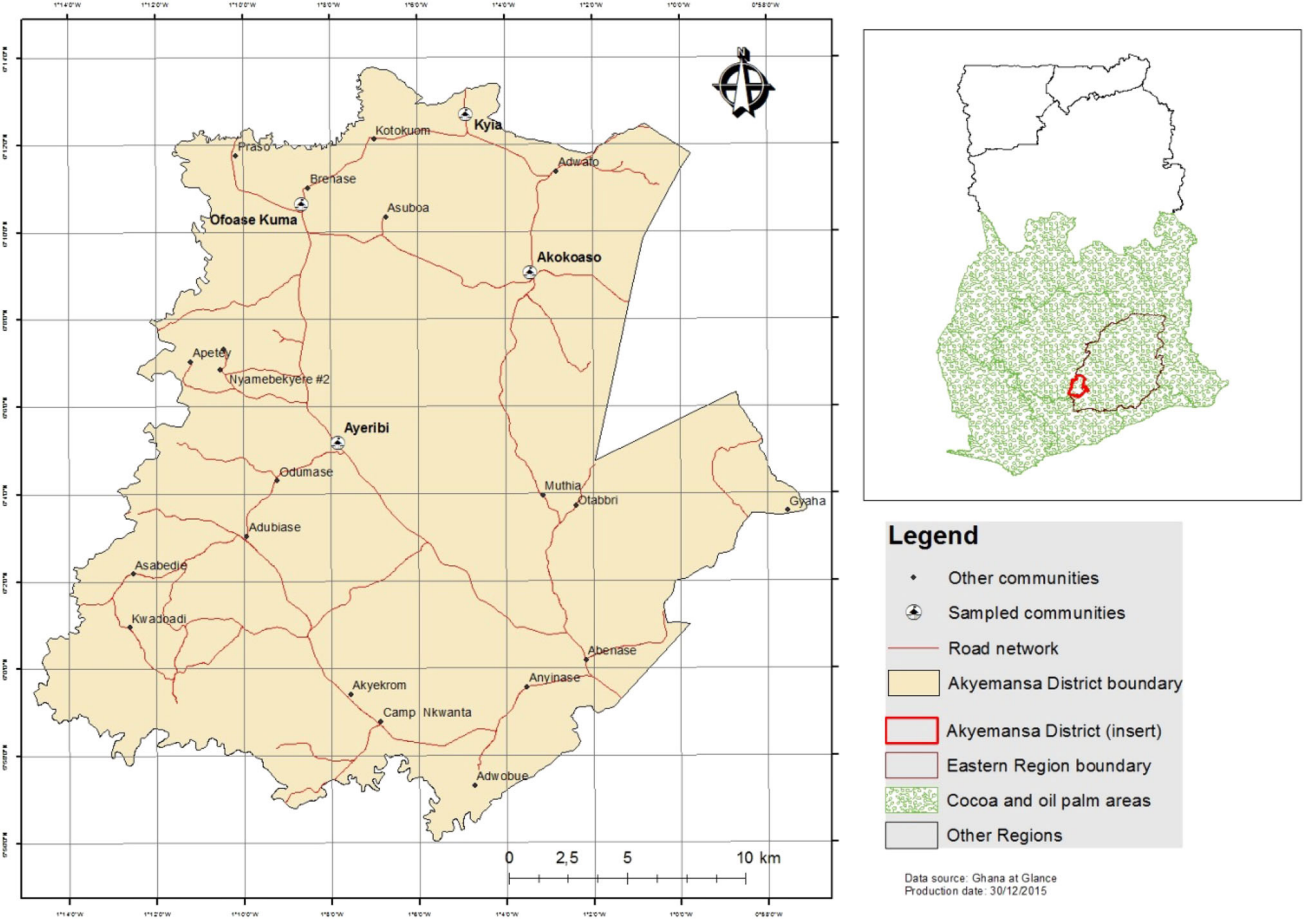
Location of the study area

This explorative study was carried out in four villages in the Akyemansa District (Fig. 1)—Kyia, Ofoase Kuma, Akokoaso, and Ayeribi—in the southwestern part of the Eastern Region of Ghana. The district covers an area of 667 square kilometers in Ghana’s semi-deciduous rain forest zone. It has a semi-equatorial climate with temperatures between 22° and 33° C and annual rainfall between 1500 and 2000 mm that mainly falls in a bimodal pattern in a long rainy season from March to July and a minor one between September and November. The district’s major rivers are the Pra River at its western boundary and the Birim River at its southern border (MOFA 2015a).

The district’s climate is favorable for the cultivation of tree crops such as cocoa and oil palm. The majority of the district’s population (87.2%)—both males and females, and both urban and rural—make a living from farming and 85 % of those grow cocoa (Ghana Statistical Service 2014). Typically, farm sizes in the Eastern Region are small, with 77% of agricultural land holdings below 1.2 ha and 16% between 1.2 and 2 ha (MOFA 2015b). The main products cultivated are tree crops (cocoa, oil palm, citrus) and food crops (cassava, maize, plantain, cocoyam, yam, and rice), with farming done with simple implements such as a hoe and cutlass (MOFA 2015a).

The major licensed buying company (LBC) active in the district is the Produce Buying Company Limited (further indicated as PBC). Having long been the unique purchasing subsidiary of Cocobod, PBC had a monopoly over cocoa purchasing until 1992, when other LBCs were also admitted. Not surprisingly, PBC remains the largest buyer in Ghana and is present in every cocoa-growing community. Its role in the Eastern Region is the mere buying of cocoa, and it was therefore selected as an example of conventional VCC.

Another major LBC in the district is Armajaro Ghana Limited (AGL), commonly referred to as Armajaro. This company, part of the multinational Ecom Agroindustrial Corp Ltd with headquarters in Switzerland, was selected as an example of advanced VCC due to it, in addition to buying cocoa, being engaged in sustainability and on-farm biodiversity enhancement programs as well as community activities with thousands of farmers (IFC 2011). Armajaro’s activities to boost farmers’ income include the distribution of implements and training in business and sustainable production skills (Modern Ghana 2011).

### Data Collection Methods

This study employed a comparative case-study design, with a view to exploring the differences between conventional and advanced VCC. Purposive sampling was used to select four villages where both PBC and Armajaro were present. Local purchasing clerks were sought out as key gatekeepers, being the direct link between farmers and buying companies. The purchasing clerks’ knowledge of their respective company history and the ability to readily contact farmers were considerations in the selection of locations. Snowballing was then employed to gain access to farmers through purchasing clerks, focusing on those selling to the buying companies studied. Convenience sampling was subsequently used to select farmers within conventional VCC, and purposive sampling to ensure that three lead farmers who were successfully integrated into the advanced VCC were included as respondents.

Mixed methods were used to collect the data. Qualitative observations throughout the fieldwork provided both general context and specific insights into existing relationships and processes within VCC. Main activities observed included the maintenance of a cocoa farm, the selling of cocoa beans, communication between buying companies and farmers, and self-provision of food. The regular observations of a research assistant have been vital in this process. Being native to Ghana and fluent in the local language Twi gave greater insight into the observations made by the principal researcher. Moreover, he built relationships with farmers, and made use of informal conversations to add insight and triangulate findings as the research progressed.

With a view to providing contextualization and the relative importance of cocoa farming in the study area, a baseline survey among 148 cocoa farmers was completed through which both qualitative and quantitative data was gathered on tree crop farmers’ characteristics, assets, market orientation, livelihood portfolios, and food security. Here too, purposive sampling was used to specifically target cocoa and oil palm farmers and to ensure that female cash crop farmers were also included. The latter are usually under-represented in surveys due to men being considered the head of the household, hence efforts were made to increase their visibility. Convenience sampling was subsequently used targeting all cocoa and or oil palm farmers within reach during the survey period.

An additional 30 participants (10 female and 20 male cocoa farmers, see Table 1) each completed both a semi-structured interview and an adapted survey, which sought greater understanding of the impact of VCC on livelihoods. The interviews focused on key concepts (Bryman 2008), while allowing for flexibility to reflect on evolving points of interest and a tailoring of questions to suit individual situations. This flexibility was vital because there was still great heterogeneity seen in farmers, despite fairly strict selection criteria, which included (i) the location of the farm (ii) the preferred LBC and corresponding location and (iii) the recent sale of cocoa to the respective LBC. Main topics always centered on the farmer’s experience, including crops they grew and practices they followed. Initial interviews asked about practices that farmers had learnt through VCC and the nature of interactions with the buying companies in terms of social, human and natural capital. As patterns emerged, this basic structure was maintained, but with a greater emphasis placed on exploring the relationship with the buying company. Questions explored how farmers’ relationship with their buying company could help in times of need, what difficulties they encountered within this relationship, and what general difficulties they experienced whilst growing cocoa. This allowed us to delve deeper into the impact of VCCs on farmers’ human, social, and natural capital.

**Table 1:** Respondents and their locations

	Interview respondents (*N* = 30)		Focus group participants (*N* = 18)	
Village	PBC (*n* = 15)	Armajaro (*n* = 15)	PBC (*n* = 9)	Armajaro (*n* = 9)
Ofoase	5	4	5	1
Kyia	6	4	4	3
Ayirebi	–*	7	0*	6
Akikwaso	4	–*	–*	–*

^*^During fieldwork the situation of the PBC contact in Ayirebi changed, meaning that it was necessary to interview farmers from Akikwaso as replacements. This meant the research was not able to include them in the focus group in Ayirebi

To assess the impact of VCC on farmers’ assets Likert scales were used, which added a quantitative dimension to the qualitative interviews (Allen and Seaman 2007). Respondents ranked the perceived impact of VCC and the relationship with the LBCs upon the most targeted assets (i.e., social, human and natural capital) as very negative, negative, neutral, positive or very positive. The focus on perceptions rather than actual impacts was a deliberate choice made from a critical realist perspective from which it is argued that there is no single truth, but that multiple accounts may exist and that the role of the researcher is to reveal these multiple subjective realities (Guba and Lincoln 1994). The economics of wellbeing (Pouw and McGregor 2014) acknowledges the cognitive or subjective dimension of wellbeing (i.e., people’s subjective assessment of and satisfaction with their quality of life) as a wellbeing dimension in its own right.

Focus groups in three communities were held (Table 1), giving the opportunity to both triangulate the research findings with communal discussion and gain further insights. The first half of each focus group was very structured, aiming to facilitate discussion between participants. Participants were asked to first identify all actors within the value chain, before discussing their respective influence on farmers’ livelihoods. This opener consistently sparked debate, encouraging an atmosphere where individuals were happy to share their thoughts. Separate tasks were then used to discuss initial findings, including (i) the practices followed and their impact, (ii) the reasons farmers had for choosing an LBC, (iii) what options farmers had if they were in need of money or assistance, and (iv) how farmers made the choice between growing cocoa and other crops. The groups discussed different aspects of cocoa farming and enabled participants to challenge each other’s points of view and to bring up their own topics for discussion (Bryman 2008). Findings were explicitly confirmed before discussions were encouraged through ranking/level of importance exercises related to previous interview questions. Vignettes, which use fictional individuals and situations to provoke comments from respondents, were also employed. These put farmers in hypothetical situations, encouraging comments and debate on individuals’ judgments, and the merits of different approaches or responses (Barter and Reynold 1999).

Finally, key respondent interviews were held with various stakeholders affiliated with PBC (*n* = 2), Armajaro (*n* = 2), UTZ Certified (*n* = 1) and Cocobod (*n* = 2) to provide a range of perspectives on the functioning of the VCC. These were less structured than farmer interviews, enabling areas of interest to be explored in depth. In practice this meant respondents were encouraged to provide a holistic view of their role, gaining information on respondents’ areas of expertise and opinions.

### Data Processing

Once data was collected, a qualitative analysis was conducted through Atlas-ti, allowing codes to be generated, applied and evaluated for common trends. This research used predetermined codes to allow analysis to focus on the theoretical framework and its operationalization (Boeije 2010), adding additional codes where needed. Quantitative analysis was performed in SPSS, with basic descriptive statistics used to describe the current context within which cocoa farmers work and to analyze effects of VCC on farmers’ capitals. To assess the extent to which advanced VCC aligns with a landscape approach we used five design principles adapted from Sayer et al. (2013) (Ros–Tonen et al. 2014).

### Limitations of the Research

Internal validity (c.f. construct validity) is generally a strength of qualitative research due to the potential for repeat interaction between researcher and respondents (Bryman 2008). Questions that directly probed the reasons behind livelihood changes, and the involvement of a research assistant familiar with the local language and context, were instrumental in this, as were the validation sessions with focus groups and inclusion of multiple chain actors in the analysis. External validity or generalizability of this study, as for all studies with a major qualitative component, is however limited due to the small sample size and the limited geographical area where the case study was carried out. Triangulation with other methods (baseline survey, focus groups) and description of the context partly compensate for this.

Additional limitations are (i) limited contextual and cultural understanding of the non-Ghanaian researcher and the risk of having missed nuances because of reliance on translations from the local language, Twi, (ii) a sample bias inherent in purposive sampling and snowballing, (iii) the presence of a buyer of either company during the focus groups, which may have prevented that the farmers spoke freely, and (iv) a lack of reliability of self-reported farm sizes. Despite these constraints, the study meets the criteria proposed by Yardley (2015) concerning (i) sensitivity to context, (ii) commitment and rigor, (iii) transparency of research methods and coherence of argument, and (iv) importance for theory, respondents, and practitioners. The clear description of methods ensures that the study can be replicated, thus guaranteeing external reliability. Internal reliability (i.e., inter-observer consistency) was achieved by constantly checking observations and interpretations with the local research assistant and third author, and through respondent validation and triangulation in focus groups.

## Results

### Actors and Institutional Arrangements

#### The demand side: Cocobod and licensed buying companies

Whilst Cocobod dictates the conditions for VCC, the LBCs in cocoa-growing communities are farmers’ main point of entry to the cocoa value chain. These companies are tasked with obtaining cocoa of a minimum standard, whilst paying a minimum price of 350 GHC (Ghana cedis) (81 USD)^1^ per bag (62.5 kg) to farmers, followed by bagging and delivery to Cocobod (World Bank 2013). Where multiple buyers exist, it is not uncommon for farmers to have relationships with multiple buyers, as a contract is not required. Buyers, then, have incentives to develop relationships with farmers to ensure a stable cocoa supply, for instance by acting as a source of credit.

In the communities studied, PBC held a unique position, seen as a long-standing, trustworthy buyer of cocoa. It did not offer farmers any additional scheme (e.g., certification) that would allow them to add further value to their cocoa.^2^ The role vis-à-vis the farmer of the representatives of PBC, the purchasing clerks, is to do no more than checking the quality of cocoa and give due payment. The cocoa proceeds along the chain, first to district offices that provide the clerks with funds to buy cocoa, then onto Cocobod which pays the producer price and an additional buyers margin, before the cocoa is finally checked again for quality and exported at the Free On Board price (Kolavalli and Vigneri 2011). Beyond this, there is no obligation for PBC to provide support to farmers, although they are often involved in distributing government-supplied inputs. However some clerks do choose to provide credit and organizing input provision for farmers.

Armajaro, the second licensed buying company studied, provides an example of advanced VCC, and how the value chain has been “upgraded” through concentrating on developing relationships, emphasizing mutual benefits and providing transparency to consumers through private certification. Armajaro is the third largest buyer of cocoa in Ghana. As with PBC, purchasing clerks are present in communities, and minimum standards of cocoa must be met. However, Armajaro has taken the additional step of promoting UTZ certification, requiring additional standards to be met by both farmers and Armajaro with regard to water and biodiversity conservation, pollution control, waste management, and increasing climate resilience (e.g., planting shade trees) (UTZ Certified 2014, 2015). As a consequence, Armajaro is able to claim an additional price premium on the world market, a proportion of which is passed to farmers in the form of a bonus on top of the minimum price, equal to 15 GHC (3.47 USD) per bag. This gives rise to a need for greater support and monitoring of farmers’ activities, leading to greater interaction with farmers and increasing their integration into the value chain. In addition to purchasing clerks, Armajaro employs a regional commercial officer who supports farmers, administers training, and aids farmers in procuring inputs for their farms. “Lead farmers”, chosen for their exemplary farming practices and strong community connections, are also used to help assist in the organization of farmers at the local level.

Buyers’ motivations for this increased integration come, first, from growing consumer concern with ethically sourced products. UTZ certification allows Armajaro to show that it has achieved a level of sustainability and working conditions above that of competitors, thus earning a premium on the world market. Second, buying companies are sensitive to supplier failure, and need to mitigate this through preventative measures in relation to the health of both farmers and their cocoa (Laven 2010). In tandem, advanced VCC promises to achieve both higher prices and higher yields of sustainable cocoa, benefitting both farmers and buyers alike.

#### The supply side: characteristics of the cocoa farmers

**Fig. 2:**
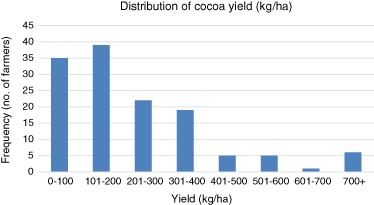
Distribution of farmers’ cocoa yield (*N* = 132)

The baseline survey showed that cocoa farmers are predominantly small-scale. Due to the observation of consistent skews in the data, we shall present the mean with the median for each variable, which is less susceptible to extreme values and which, we believe, gives a better view of the typical characteristics of Ghanaian cocoa farmers.^3^ The mean amount of land devoted to cocoa was 3.26 ha, with a median of 2.80 ha and 75% of farmers having 5 ha or less. This is considerably more than the aforementioned figures provided by the Ministry of Food and Agriculture (MOFA 2015b), but can be due to errors in self-reporting. The household head was a full-time cocoa farmer in 73% of cases, with a further 57% of spouses also indicating that cocoa was their full-time occupation. Mean reported yields were 237 kg/ha, below the average of other studies within Ghana that have previously reported an average of 350–400 kg/ha (Terazono 2014; Asare 2016). However, the large standard deviation of 199 shows a large diversity in farmers’ yields (Fig. 2). This can be due to the variance in quality of land, farming practices, or simply individuals’ inability to estimate the size of land they devote to cocoa. The value of 191 confirms the low productivity in the study area. Despite this low productivity, the baseline highlights the importance of cocoa for incomes in the area. Mean income was 1850 USD, slightly below Ghanaian gross national income per capita of 1910 USD. However, the median was significantly lower, at USD 1389. Of this, our baseline found 75% of income is reliant upon crops in general. Previous studies have indicated a high dependence on cocoa for income, with a reported 67% of income from cocoa alone (Kolavalli and Vigneri 2011). Our baseline echoes these findings, with 76% of cocoa farmers reporting it as their most important crop and 23% putting it as second.

Despite the importance of cocoa, access to alternative markets is key for small‐scale cocoa farmers in Ghana who typically cultivate multiple crops for sale or subsistence. Oil palm, another cash crop, was reported as either the most or second most important crop for 67% of farmers. Subsistence farming is also common and all farmers reported at least a home garden, where food crops are grown. The main crops grown in the study area were cocoa, oil palm, and food crops, with each three providing different benefits to farmers. Cocoa guarantees farmers a market with a guaranteed price, while oil palm has two main marketing routes. The Ghana Oil Palm Development Company, using out-grower schemes, offers a given price, relative to world prices. Local markets are also available, mainly through female traders and processors. On local markets, prices are negotiated per transaction and can be volatile. A third option for those with the right knowledge is to process palm oil themselves, but this is less prevalent in the study area. Finally, food crops are grown for both home consumption and local markets. Prices are negotiated and can vary significantly throughout the year.

Although an analysis of the surrounding culture, societal norms, and accepted hierarchies that impact farmers’ behavior is beyond the scope of this paper, we found it useful to consider Hofstede’s (1984) cultural dimensions to give some contextual understanding of the findings. Consistent with Hofstede’s findings, we found that Ghanaian culture expects and accepts hierarchies of power; puts high importance to group and family ties; is relatively risk-averse; trusts past tradition rather than seeking societal change; and prefers short-term gains over long-term investments (Hofstede Centre 2015).^4^

### Perceived Effects on Farmers’ Social, Human, and Natural Capital

**Fig. 3:**
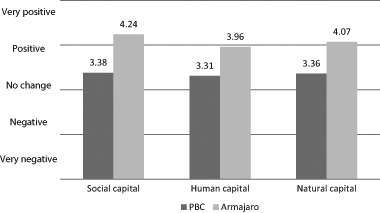
Farmers’ appreciation of effects on social, human and natural capital (*N* = 30)

Figure 3 clearly shows that farmers within advanced VCC feel more positive about the effect of their interactions with their LBC on social, human and natural capital. Below we elaborate on the differences for each of these capitals.

#### Social capital

Scores for farmers’ appreciation of effects on social capital were 3.38 and 4.24 for conventional and advanced VCC respectively. In general, there is limited communication among farmers. All cocoa farmers automatically become members of a national union, but this does not affect daily life nor enhance formal meetings between them. Rather than participating in union meetings, the instant benefits of which are unclear to them, farmers expected to have elected executives and leaders to act on their behalf. This aligns with the cultural context of strong hierarchical relationships (Derkyi 2012). Farmers report seeking informal advice on chemicals, practices, and yields from other farmers they see doing well, but, in general, there are no additional provisions for communication beyond the community.

Hence the differences in perceived strength of social capital can be attributed mainly to differences between the companies’ approaches toward organizing the farmers. PBC has no formal way of contacting the farmers; information is generally spread through word of mouth and communication occurs only at the point of sale focusing primarily on the quality of beans. With limited avenues for voicing opinions at formal meetings, the effect of conventional VCC on farmers’ social capital is therefore constrained.

Communication between farmers and Armajaro is distinctive in the advanced VCC. Armajaro relies on purchasing clerks and identified “lead farmers” to formally organize farmers. Meetings are regular, with Armajaro providing training, advice and certification groups where farmers discuss challenges in meeting certification requirements. Attendance is encouraged with small incentives in the form of food or inputs, while meetings are also aided by the commercial officer committing to regular trips to communities and certified farms. This increases farmers’ social capital, and their ability to express concerns to Armajaro. The regular meetings and word-of-mouth promotion among farmers about certification translate into more farmer-to-farmer communication, hence bonding capital within the community (i.e., an increase in ties among a fairly homogenous group) (Putnam 2000; Woolcock 2001). This occurs both within Armajaro, but also with farmers outside the VCC, resulting in increased bridging social capital (i.e., network ties between more distant groups operating at similar levels) (Woolcock 2001; Szreter and Woolcock 2004). However, there remains disconnect between farmers and actors further up the chain (linking social capital or network ties between groups and institutions across different situations and levels of scale) (Woolcock 2001; Szreter and Woolcock 2004).

#### Human capital

Human capital development (mainly training and health aspects) also scored higher among those engaged in advanced VCC (3.96) than among those within conventional VCC (3.31). These scores refer only to cocoa; there was very little difference in farmers’ knowledge gained on other crops (not displayed in Fig. 3). This means that no effects of advanced VCC on food security can be expected other than through increased incomes.

Within conventional VCC, there is limited knowledge sharing between PBC and farmers. On occasion, there are meetings to discuss best practices for fermenting and drying cocoa beans, but these are not sufficient for producers who suffer from diseases on their farms and/or decreasing yields, or for those who use toxic chemicals such as DDT. Outside of VCC, farmers occasionally receive information and training from extension officers from Cocobod or the MoFA. Sessions organized by extension officers visiting a community increased knowledge of practices such as regular pruning, weeding and the use of fertilizer, but farmers appeared not to be aware of the justifications for these practices, which limited the impact of such training.

Health is a consistent concern for farmers, and older farmers report a number of health-related issues. During times of ill health, it can be difficult for farmers to harvest, and there is no formal provision in conventional VCC to help farmers remain healthy. In one community, the local buyer was able to organize labor to work for farmers when they were too ill, but this was a welcome exception rather than the norm. For human capital, in terms of both knowledge and health, there are needs that were not met within conventional VCC, and limited avenues for farmers to address these concerns.

Within advanced VCC farmers are trained on certification standards and how to meet them. This involves agricultural practices as well as health and safety guidance, aimed at growing cocoa in a sustainable way. Farmers’ knowledge of agricultural practices is increased, including pruning, the spacing of trees, cocoa nurseries, non-toxic fertilizers and the correct application of these fertilizers. In addition, farmers engaged in advanced VCC attend group meetings, providing increased opportunities to receive advice and enhance their human capital. However, training not only refers to sustainable farming skills, but also to risks related to the unprotected use of pesticides, which can reportedly lead to impotence, blindness, and even death if exposed over a long period. Selected farmers in communities are trained and given protective equipment, along with health checks every 3 months. Other community members can then approach the trained individual when their cocoa requires spraying, avoiding the risks that are inherent in the handling of chemicals.

The impact of training is often felt within a season and can increase yields from a common yield of 2–3 bags per acre to between 6–8 bags per acre. This increase, in addition to the higher price of each bag, greatly increases farmers’ income, resulting in an average income per hectare of 1536 GHC (355 USD) for farmers in advanced VCC vs. 941 GHC (217 USD) for those in conventional VCC. It is also clear that farmers appreciate the rationale behind the practices they follow, with a detailed understanding of how increased biodiversity, replenished soil, and increased air circulation all contribute toward increased cocoa yields and long-term sustainability. Rather than having practices forced upon them, farmers show that they understand the rationale behind these practices.

#### Natural capital

Consistent with the findings on training in sustainability standards and practices, farmers in conventional VCC did not take any clear sustainability measures to preserve their natural capital beyond adding fertilizers. Advanced VCC, in contrast, aims to ensure sustainable cocoa supply through the implementation of certification standards. Practices such as planting shade trees, avoiding harmful chemicals, refraining from hunting and strict rules on encroaching protected areas (UTZ Certified 2014) assumedly enhance natural capital and farmers indeed perceive such improvements (Fig. 3): the average score of perceived effects on natural capital is 3.36 among farmers engaged in conventional VCC vs. 4.07 for those within advanced VCC.

Changing their agricultural practices directly translates into positive productivity effects: the survey data shows significant differences in yields between farmers engaged in conventional and advanced VCC, with average yields differing as much as 168 kg/ha (sd = 53.4) and 263 kg/ha (sd = 131.36), respectively. The standard deviations of each sample are high, however, which reflects the heterogeneity among farmers in general. The larger standard deviation within the sample of farmers within advanced VCC is potentially a sign that some farmers are further along the path of certification than others, with the range at higher levels of yield much greater than those within conventional VCC. The difference between the two groups seems to confirm evidence from other studies that certification programs mainly target better-off farmers or “low-hanging fruits” (SUSTAINEO 2013; Barrientos 2016). Armajaro, for instance, requires farmers to have a minimum farm area of 2 acres in order to qualify for the certification program (PC Armajaro Kade, pers. comm.).

## Discussion and Conclusion

### Actors and Institutional Arrangements

In terms of actors and institutional arrangements, VCC in Ghana’s cocoa sector operates within a well-regulated cocoa market within which Cocobod has significant influence over actors. LBCs have developed different strategies, resulting in examples of both conventional and advanced VCC. In conventional VCC, with interactions between actors being limited to selling-buying, checking the minimum standard and paying the minimum price set by Cocobod, it is hard for buyers to assess the efficiency of farming practices, and farmers, in turn, are not encouraged to approach their buying company for advice. In contrast, within advanced VCC, there is regular interaction with and support to farmers to achieve increased and more sustainable cocoa harvests. The training provided—often involving NGOs as trainers or auditors—is not revolutionary, and is known to many within the cocoa sector, but requires communication that encourages farmers to make investments in their cocoa production. Within advanced VCC, purchasing clerks not only act as intermediaries between farmers and the cocoa-buying company, but also as brokers of knowledge, inputs, and resources (Kooijmans 2016; Le Guillouzic 2016).

### Effects on Farmers’ Capitals

With dedicated training for farmers and follow-on support, farmers engaged in the studied advanced VCC are able to achieve greater yields of cocoa, use less harmful inputs, and benefit from the subsequent increases in income and health (financial and human capital). Higher yields and healthier farmers, in turn, mean that Armajaro benefits from an increased and sustainable supply of cocoa beans.

With such differences between conventional and advanced VCC, one wonders why not all farmers are engaging with Armajaro. The main explanatory factor is the cultural tradition of selling to PBC, which was the only buying company in Ghana since Independence until the cocoa market was partially liberalized in 1992 (Kolavalli and Vigneri 2011). This fits within Ghana’s cultural profile (Hofstede 1984; Hofstede Centre 2015) where farmers are relatively risk-averse and trust past tradition rather than seek societal change. Farmers remain loyal to the buying company to which their parents sold and with which they developed a long-standing relationship. Against this background, they are not inclined to explore further options, despite their social and human capital needs being largely unmet. Hence they are often unaware of the incentives available for certified cocoa and the productivity gains or are simply not motivated to change agricultural practices. There may also be an economic incentive to stay with PBC, as the company tends to have a more flexible policy with regard to advance payments than Armajaro, thus enabling farmers to access credit when in need.

### Potential for Landscape Approaches

Although the Armajaro VCC was not explicitly designed as a landscape approach, several of the principles and enabling conditions for a landscape approach (Ros–Tonen et al. 2014; see also Sayer et al. 2013) apply to the collaboration (Table 2). The overview shows that advanced VCC embraces an integrated approach with potentially multiple positive outcomes at the landscape scale through the promotion of sustainable agricultural and conservation practices embedded in the certification standard. In farmers’ perceptions, this boosted their social, human and natural capital. Positive effects on yields have also been documented elsewhere (Blackman and Rivera 2010; Jones and Gibbon 2011; SUSTAINEO 2013). Conservation effects are harder to measure due to the mismatch between the focus of certification programs on the farm level and biodiversity processes occurring on a landscape scale (Tscharntke et al. 2015). This also applies to effects on climate resilience and landscape management in general.

**Table 2:** Potential of advanced VCC as an entry point for implementing landscape approaches

Principle/enabling factor^a^	Meaning	Example in advanced VCC	Potential for a landscape approach
Integrated approach (Sayer et al. 2013)	∙ Integrated conservation and development aims∙ Integration of environmental, economic and social and ecological objectives	∙ Targets both increased cocoa production, livelihood improvement, and sustainability aims	∙ Integrated approach aligns with objectives of a landscape approach, but certification standards target the farm, not landscape level.
Multi-stakeholder negotiation (Sayer et al. 2013)	∙ Negotiated goals∙ Shared vision and consensus about desired changes∙ Negotiation of conservation-development trade-offs	∙ Frequent meetings with farmers, but limited negotiation procedures in place.	∙ Negotiation within VCC is mainly centered on access to credit and inputs. This study provides no evidence that advanced VCC can provide a way toward negotiating land use and trade-offs; not all landscape stakeholders are involved.
Polycentric governance (Nagendra and Ostrom 2012)	∙ Hybrid institutional arrangements∙ Rights, responsibilities, and benefits clear to all∙ Legal options for self-organization	∙ Armajaro makes use of lead farmers and traditional leaders in the villages, thus blending old and new institutional arrangements.∙ Rights, responsibilities, and benefits are clearly stipulated.∙ Options for self-organization are limited; Armajaro takes the lead.	∙ Negotiated land use at landscape level requires the involvement of traditional authorities who determine access to land, and collaboration with the Forestry Commission which holds jurisdictional power over forest reserves and naturally regenerated trees in off-reserve areas.∙ Rights and responsibilities refer mainly to cocoa production. Parties in the VCC have too limited jurisdictional power to affect or negotiate land use at the landscape level.∙ Self-organization remains limited because (i) hierarchical relationships prevail, (ii) transaction costs of bringing stakeholders together are high, and (iii) this is not in the interest of or facilitated by the buying company
Continual learning (Pahl–Wostl 2009; Gupta et al. 2010; Sayer et al. 2013)	∙ Single-loop learning: improving routines∙ Double-loop learning: reframing assumptions∙ Triple-loop learning: transforming underlying norms and values∙ Institutional memory (learn from monitoring and evaluation)	∙ Single-loop learning occurs through changed agricultural practices; to some extent, double-loop learning through increased awareness of the rationale behind these practices. This may eventually lead to third-loop learning, e.g., with regard to sustainability practices.	∙ Understanding the rationale underlying the proposed agricultural practices, combined with evidence of gains (increased yields and a premium price), appeared to be important incentives to change agricultural practices. This provides an entry point for continual learning.
Adaptive capacity (Dietz et al. 2003 ; Armitage 2005; Berkes 2009; Gupta et al. 2010; Sayer et al. 2013)	∙ Being prepared for change∙ Willingness to engage in collective decision-making and share power∙ Accept a diversity of solutions, actors, and institutions∙ Room for autonomous change	∙ Farmers are to some extent prepared to change agricultural practices, while licensed buying companies in advanced VCC are prepared to invest in the relationship with farmers. However, the willingness to engage in collective decision-making, share power, accept a diversity of solutions and create room for autonomous change remains limited at both sides.	∙ The local cultural context, dominated by values of loyalty and hierarchical relationships, and being risk averse, plays an important role in farmers’ preparedness to change. Evidence of increased yields and a price premium, however, act as effective incentives. Licensed buying companies have limited interest in accepting a diversity of solutions, actors, etc.: their main interest is securing supplies and tie farmers to their firm, thus limiting the scope for autonomous change.
Social capital (Putnam 2000; Woolcock 2011; Pretty 2003)	∙ Bonding social capital: relations of trust, reciprocity, and exchange; common rules, norms and sanctions∙ Bridging social capital: horizontal linkages between different groups (e.g., communities)∙ Linking social capital: vertical linkages with external organizations and agencies	∙ Huge effect on bonding and bridging social capital; limited effect on linking social capital.	∙ Regular communication instills trust and long-standing relationships. Frequent interactions between buyer and farmers also stimulate interactions among farmers. As such advanced VCC provides an entry point for mobilizing small-scale farmers. Constraints to building linking social capital, however, limit the potential to use advanced VCC for implementing a landscape approach.
Bridging organizations (Berkes 2009; Cundill and Fabricius 2010)	∙ Long-term facilitation and leadership	∙ Purchasing clerks act to some extent as a bridging organization by providing access to markets, premium prices, and training. They occasionally also provide access to credit, and can facilitate the provision of both inputs and labor.	∙ Farmers attribute responsibilities to their direct contact (their purchasing clerk) that are beyond their control and influence. Implementing a landscape approach requires additional partnerships and an impartial brokering organization.
Long-term funding (Cundill and Fabricius 2010)	∙ Long-term financial security or commitment	∙ As long as there is a stable cocoa market with a guaranteed minimum price, and buying companies striving for sustainable supplies, VCC in Ghana has a sustainable financial basis.	∙ There are no signs of financial limitations to advanced VCC in Ghana’s cocoa sector hence there is potential for a stable entry point for an integrated landscape approach.

^a^ Categorization based on Ros–Tonen et al. (2014), partly based on Sayer et al. (2013).

However, with VCC increasingly extending “beyond the chain”, there seems to be scope to align advanced VCC with a broader landscape approach which targets the companies’ sourcing area. Sustainable sourcing and creating shared values strategies provide incentives for companies to engage in such approaches. Synergies between advanced VCC and landscape approaches exist based on (i) an integrated approach toward sustainable production, biodiversity conservation, and (in some cases) “climate smart” cocoa landscapes (Kissinger et al. 2015), (ii) a focus on continual learning, (iii) strong social capital, which is conducive to a multi-stakeholder approach, and (iv) a stable relationship with small-scale farmers.

We see three areas where advanced VCC can strengthen the practical implementation of a landscape approach. The first is through providing economic sustainability that comes from a commercially driven collaboration, generating mutual benefits for companies, conservationists, and local communities. Second, VCCs targeting international export markets demand active participation from local communities, local and national businesses, and local-to-national government, and landscape approaches can use these existing linkages to enhance landscape governance. Third, landscape approaches are only successful when there is buy-in from local communities. Existing VCC “beyond the chain” allows practitioners to develop a bottom-up approach and identify local-level priorities which can be aligned with those of landscape approaches.

It is clear, however, that advanced VCC such as the one analyzed in this paper is not a landscape approach in the sense of negotiating land use and trade-offs. The primary incentives for both farmers and buyers to invest in the VCC are economic—access to markets and inputs, increased yields and a premium price for the farmers; sustained supplies and a profitable niche market for sustainably produced and fairly traded cocoa for the buying companies. Rather than being negotiated, the change logic is determined by the buying company and its partners at the global level (e.g., UTZ) and options for self-organization are limited.

A landscape approach requires the engagement of actors with the jurisdictional power to decide on land allocation and use—in Ghana traditional authorities (who hold land in custody for the communities) and the Ghana Forestry Commission (which decides on forest reserves and naturally generated timber trees on farming land). Such alliances were not observed in this case, but have been described for agribusiness Olam International when it initiated a landscape approach in West Africa’s cocoa sector (Kissinger et al. 2013; Brasser 2013). The company negotiated better tenure arrangements with traditional authorities and concession holders and engaged in a national multi-stakeholder platform involving the Ghana Forestry Commission to negotiate the integration of cocoa farming in carbon schemes. Such examples are however still scarce and require deliberate steps in that direction—steps that probably require a brokering or bridging organization to materialize.

In conclusion, the scope for negotiation and self-organization still seems to be too limited for advanced VCC to develop into a landscape approach by itself. There is, however, scope to use advanced VCC as an entry point for implementing a landscape approach. Aligning advanced VCC with a landscape approach would require (i) extending the scope of certification to the landscape scale through group certification and “landscape labeling” (Tscharntke et al. 2015; Kissinger et al. 2015), (ii) partnering with other landscape actors to address objectives beyond the scope of VCC and jurisdictional powers of its actors, and (iii) a bridging organization which mediates between the actors involved and enhances their linking social capital. Further (action) research could shed light on the terms of engagement of the various actors in such approaches and options to increase self-organization of the farmers involved.

## Footnotes

1 Based on the exchange rate of USD 1: GHC 4.32 on 15 June 2015 when the data was collected.

2 Trial certification schemes are being run by PBC in other regions of the country, predominantly the Western and Brong–Ahafo regions.

3 In addition, we excluded two outliers from the calculations, with values that cannot even be reached with hybrid cocoa under ideal experimental conditions.

4 Although Hofstede’s cultural dimensions of power distance (acceptance of unequally distributed power), uncertainty avoidance, individualism, masculinity and long-term vs. short-term orientation have been criticized for resulting in stereotypes, there is a broad literature confirming their relevance at national level (e.g., Soares et al. 2006). More recently, attempts have been undertaken to develop a five-dimensional scale that assesses Hofstede’s dimensions at the individual level, allowing for more diversification (Yoo et al. 2011).
